# The Relationship of Malnutrition With Cognitive Function in the Older Chinese Population: Evidence From the Chinese Longitudinal Healthy Longevity Survey Study

**DOI:** 10.3389/fnagi.2021.766159

**Published:** 2021-11-22

**Authors:** Boran Sun, Yihao Zhao, Wenli Lu, Yongjie Chen

**Affiliations:** Department of Epidemiology and Statistics, School of Public Health, Tianjin Medical University, Tianjin, China

**Keywords:** geriatric nutritional risk index, malnutrition, cognitive function, Chinese elderly, linear mixed-effects model

## Abstract

**Background and Objective:** Few studies have explored the relationship between malnutrition measured by the Geriatric Nutritional Risk Index (GNRI) and cognitive performance. This study aimed to investigate the association of malnutrition with cognitive function in the Chinese population.

**Methods:** It was a prospective longitudinal study and used three waves of the Chinese Longitudinal Healthy Longevity Survey (CLHLS) data in 2011–2012, 2014, and 2017–2018. Participants aged 60 years or older without mental illness and cerebrovascular diseases were eligible. The GNRI was used to assess nutritional status as follows: normal nutrition (a GNRI > 98), mild malnutrition (92 ≤ a GNRI ≤ 98), and moderate-to-severe malnutrition (a GNRI < 92). Cognitive performance was evaluated by the Mini-Mental State Examination (MMSE) scores. The relationship between the GNRI and cognitive function was analyzed using a linear mixed-effects model.

**Results:** A total of 1,632 subjects were analyzed, including 741 males and 891 females. Of these, 65.0, 19.4, and 15.6% of subjects were at normal nutritional status, mild, and moderate-to-severe malnutrition, respectively. After adjusting for potential confounders, participants under mild and moderate-to-severe malnutrition status have a lower MMSE score [β (95% CI): –0.95 (–1.60, –0.25) and –1.39 (–2.21, –0.57), respectively], compared with those having normal nutrition. Also, there was a linear trend in the association of malnutrition risk with cognitive function in the total population [β (95% CI): –0.74 (–1.13, –0.35)]. However, a significant association of malnutrition with cognitive function was observed only among illiterate females aged above 90 years.

**Conclusion:** This study suggested that there was a significant relationship between the GNRI and cognitive function in the Chinese elderly. Furthermore, subjects with more serious malnutrition have a worse cognitive function, especially in the oldest illiterate females. Clinicians should put more emphasis on assessing the nutritional and cognitive status of the elderly to timely intervene and prevent cognitive impairment.

## Introduction

It is estimated that more than 1.5 billion people will be aged 65 or older by 2050, accounting for 16% of the total population. Consequently, further attention should be paid to the physical and mental health of the elderly (United Nations, 2019). With the population aging, malnutrition caused by an energy imbalance between the intake and requirements has been a serious global public concern. As a previous review reported, 23–60% of the hospitalized elders were malnourished in developed countries (Agarwal et al., 2013), while 48.4% of community-dwelling elderly were identified to have a high malnourished risk in China (Kang et al., 2018). Older people with malnutrition will have increased risks of fall (Lackoff et al., 2020), frailty (Hong et al., 2019), prolonged length of stay in the hospital, morbidity, and mortality (Correia and Waitzberg, 2003; Lim et al., 2012; Leiva Badosa et al., 2017).

Meanwhile, mild cognitive impairment (MCI), as a transitional state between normal aging and dementia, has affected 10–15% of the population aged 65 and above (Anderson, 2019) and gradually became a serious public health problem among old people worldwide. Cognitive impairment is characterized by a decline in memory, attention, language, and other cognitive function beyond age (Eshkoor et al., 2015), which had imposed great burdens on family and society. Compared with those with stable cognitive function, participants with rapid cognitive decline had a 75% higher risk of death (Lv et al., 2019). The prevalence of cognitive impairment was about 16.5 and 16.8% in the European and Chinese elderly, respectively (Hou et al., 2019; Pais et al., 2020). Moreover, 12–15% of old people with MCI will develop irreversible dementia within 1 year (Hill et al., 2017). As there were still no effective disease-modifying pharmacologic treatments for dementia (Tisher and Salardini, 2019), it is crucial to identify protective factors and improve early detection for MCI.

Previous studies have demonstrated that nutritional status played a significant role in the progression of cognitive impairment in older patients with dementia or Alzheimer’s disease (AD) (Ousset et al., 2008; Santos et al., 2018; Kimura et al., 2019; Doorduijn et al., 2020). Recent researches have also illustrated that malnutrition was associated with an increased risk of cognitive decline among older adults (Hsu et al., 2019; Assis et al., 2020; Mantzorou et al., 2020; Yu et al., 2021). However, most of the studies have explored the relationship by utilizing Mini Nutritional Assessment (MNA) (Assis et al., 2020; Mantzorou et al., 2020) or Mini Nutritional Assessment Short-Form (MNA-SF) (Hsu et al., 2019; Yu et al., 2021), but not the Geriatric Nutritional Risk Index (GNRI). MNA and MNA-SF were defined as those not using biological indicators, such as albumin. While the GNRI combines two nutritional indicators, namely, albumin and weight loss. Furthermore, usual body weight was replaced by ideal body weight in the NRI formula when usual weight failed to be obtained in the elderly population. The GNRI was initially developed to evaluate the nutrition-related risk in hospitalized older people (Bouillanne et al., 2005). Until present, the GNRI could comprehensively identify the elderly under malnutrition risk and has been proved to predict morbidity and mortality (Hao et al., 2019; Xie et al., 2020). The area under the curve (AUC) of the GNRI predicting a composite of events such as all-cause death was 0.873 in the Chinese population (Zhao Q. et al., 2020). The validity and reliability of the GNRI among Chinese or Asian older adults have been reported in previous studies (Abd Aziz et al., 2019; Nishi et al., 2019; Zhao Y. et al., 2020).

Accordingly, our study aimed to further investigate the relationship of malnutrition, measured by the GNRI, and cognitive function in the Chinese elderly, based on the three waves of the Chinese Longitudinal Healthy Longevity Survey (CLHLS) data in 2011–2012, 2014, and 2017–2018.

## Materials and Methods

### Study Design and Population

Data were based on the CLHLS study. The CLHLS is a nationwide community-based prospective longitudinal study, aiming to investigate health status and factors influencing longevity in the Chinese elderly. Its baseline survey began in 1998, and seven follow-up survey waves were conducted in 2000, 2002, 2005, 2008–2009, 2011–2012, 2014, and 2017–2018, respectively (Yi, 2008; Zeng, 2012). To obtain representative information of Chinese populations at an advanced age, the CLHLS study oversampled the oldest-old and was enriched for centenarians, such as 19.5 thousands centenarians, 26.8 thousands nonagenarians, and 29.7 thousands octogenarians (Zeng, 2013; Zeng et al., 2017). The validity of the CLHLS has been widely reported elsewhere (Gu et al., 2009, 2017).

In addition, a biomedical in-depth CLHLS study was performed in the CLHLS of waves 2008–2009, 2011–2012, and 2014 in sequence. During the in-depth study, physical examinations and almost 30 biomarkers in blood and urine samples were collected by the trained medical staff of the Chinese Center for Disease Control and Prevention local network. More details of the in-depth CLHLS study have been published elsewhere (Zeng et al., 2017; Lv et al., 2019; Liu et al., 2020). The CLHLS study was approved by the Institutional Review Board, Duke University (Pro00062871), and the Biomedical Ethics Committee, Peking University (IRB00001052–13074). All participants gave written informed consent.

**FIGURE 1:**
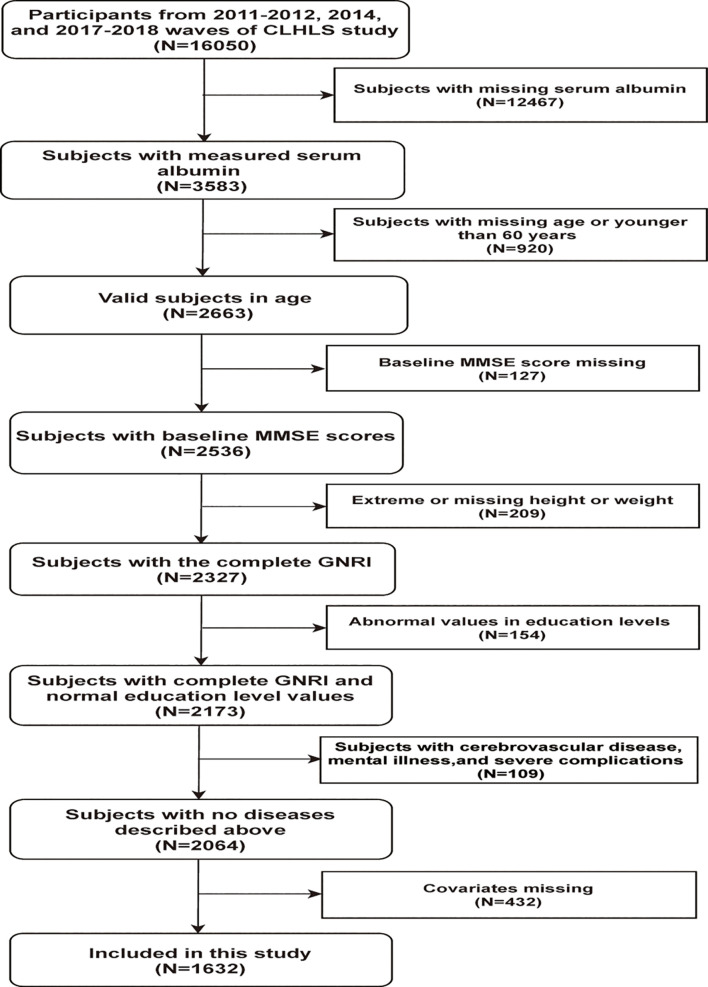
Flowchart of the study population.

In this study, we used three waves of the CLHLS data in 2011–2012, 2014, and 2017–2018. The inclusion criteria were as follows: participants aged 60 years or older; and participants with complete baseline information of the GNRI and Mini-Mental State Examination (MMSE) score. The exclusion criteria were as follows: participants with missing data in covariates; and participants with a history of intracranial tumor, craniocerebral injury, acute infection, mental illness, and cerebrovascular diseases, which were self-reported. Eventually, 1,632 eligible participants were included. The detailed information is presented in Figure 1. If the baseline survey was conducted in the wave 2011–2012, participants were followed up consecutively in the 2014 and 2017–2018 waves. Otherwise, if the baseline survey was only in the 2014 wave, follow-up was conducted in the wave 2017–2018. Therefore, there is no overlap in our final analyzed population.

### Data Collection

The baseline data were collected using a standardized questionnaire in a home-based interview by trained investigators in 2011–2012 and 2014 waves. The information included (1) sociodemographic characteristics such as age, sex (male/female), ethnicity (Han/other), residence (urban/rural), marital status (married/other), education level (no schooling/some schooling), and living alone (yes/no), (2) lifestyle-related variables such as current smoking status (yes/no), drinking status (yes/no), and regular exercising (yes/no), and (3) health status involving a self-reported history of heart disease (yes/no), hypertension (yes/no), diabetes (yes/no), and arthritis (yes/no). Participants were divided into three age groups: 60–79 years, 80–89 years, and ≥90 years.

The participants were asked to report their food intake frequency of last month of vegetables, meat, eggs, and fish. The intake frequencies of meat, fish, and eggs were recorded as “almost every day,” “occasionally,” or “rarely or never.” While for vegetables, the intake frequency was “almost every day,” “almost every day except in winter,” “occasionally,” and “rarely or never” (Shi et al., 2015). In the analysis, we classified the frequency of “almost every day except in winter” as “almost every day.” The ability of basic activities of daily living (BADL) was measured by a question: Do you need assistance in bathing/dressing/toileting/transferring/eating/continence? According to a previous study using the data of the CLHLS, a score of 0 was given if no help was needed, and a score of 1 was given if some or complete help was needed. The BADL score ranged from 0 to 6 (Zeng et al., 2017). Based on the BADL score, we divided participants into having BADL disability (BADL score > 0) and not having BADL disability (BADL score = 0).

After the interview, the anthropometric measurements were carried out to obtain height (in meters) and weight (in kilograms). All measurements were carried out three times, and the average values were recorded. Body mass index (BMI) was calculated as weight divided by the square of height.

Serum albumin was measured by trained personnel using an automatic biochemistry analyzer (Hitachi 7180, Japan; Roche Diagnostic, Mannheim, Germany) at the central laboratory at the Capital Medical University in Beijing.

### Nutritional Status Assessment

In this study, the GNRI was used to evaluate nutritional status. The GNRI is calculated by albumin and weight loss as follows: GNRI = [1.489 × serum albumin (g/L)] + [41.7 × (actual weight/ideal weight)] (Bouillanne et al., 2005). Weight loss is reflected by ideal body weight and actual weight. Different Lorentz formulas were used to calculate the ideal weight according to sex. For males, ideal weight was calculated by 0.75 × height (cm)–62.5, and ideal weight was obtained using 0.60 × height (cm)–40 for females (Matsuzawa et al., 1990). If the actual weight is greater than the ideal weight, the actual weight/ideal weight is set to 1 (Matsuzawa et al., 1990; Bouillanne et al., 2005). Participants were divided into four groups based on the GNRI as follows: normal nutrition group, a GNRI > 98; mild malnutrition group, a GNRI ≥ 92 but ≤ 98; moderate malnutrition group, a GNRI ≥ 82 but < 92; and severe malnutrition group, a GNRI < 82 (Bouillanne et al., 2005). In this study, since serum albumin failed to be collected in the wave 2008–2009, the GNRI was calculated only using the data of 2011–2012 and 2014 waves in the CLHLS. Moderate and severe malnutrition groups were combined into a moderate-to-severe group. The validity and reliability of the GNRI have been reported in previous studies (Abd Aziz et al., 2019; Nishi et al., 2019; Zhao Q. et al., 2020).

### Cognitive Function Evaluation

In each wave of the CLHLS, cognitive function was assessed by the Chinese version of MMSE, which was adapted from the international version that was proposed by Folstein et al. (1975) and validated in previous publications (An and Liu, 2016; Zeng et al., 2017; Zhang et al., 2019). In view of cultural and socioeconomic factors in the Chinese elderly, the Chinese version of the MMSE consists of 13 questions, covering 5 domains of cognitive function, namely, orientation, registration, attention and calculation, recall, and language. With total scores ranging from 0 to 30, a lower score of MMSE indicated a poorer cognitive function. More details about the Chinese version of MMSE are shown in Supplementary Table 1.

### Statistical Analysis

Continuous variables with normal distribution were presented as means ± SD. Non-normal variables were reported as median (P_25_, P_75_), whereas categorical variables were expressed as frequencies (percentages). The distributions of baseline characteristics were compared by one-way ANOVA, Kruskal–Wallis non-parametric test, and χ^2^ test across the normal, mild malnutrition, and moderate-to-severe malnutrition groups. For the primary analysis, a linear mixed-effects model was employed to estimate the effects of malnutrition on cognitive function, adjusting for age, sex, ethnicity, residence, marital status, education, living alone, regular exercising, smoking, drinking, history of heart diseases, diabetes, hypertension, and arthritis, BADL disability, and the frequencies of meat, fish, eggs, and vegetables. In the linear mixed-effects model, the MMSE scores from waves 2011–2012, 2014, and 2017–2018 were response variables, and malnutrition assessed by the GNRI was an independent variable. Furthermore, the linear mixed-effects model was stratified by sex, age, and education level. Four sensitivity analyses were conducted. First, the GNRI as a continuous variable was considered as an independent variable in the linear mixed-effects model. Second, serum albumin and BMI were used to construct linear mixed-effects models, respectively. Third, the mediation analysis was used to examine whether the association between malnutrition and cognitive impairment was mediated by hypertension and diabetes. Finally, we imputed the missing covariates using the R software package “*mice*” to evaluate the influence of missing covariates on the results. Two-tailed *P* < 0.05 was considered to be statistically significant, and all analyses were conducted using SAS 9.4 (SAS Institute Inc., Cary, NC, United States).

## Results

### Characteristics of Participants

Among 1,632 subjects, the average age was 85.57 ± 12.35 years old, and 54.6% of subjects were females. The proportion of participants in the normal nutrition, mild malnutrition, and moderate-to-severe malnutrition groups was 65.0, 19.4, and 15.6%, respectively. As shown in Table 1, the risk of malnutrition was higher in an older female with a lower MMSE score, BMI, and serum albumin concentration (*P* < 0.001). Also, subjects who have been married (*P* < 0.001), had regular exercises (*P* < 0.001), and ever attended school (*P* < 0.001) were less likely to be malnourished. Additionally, in contrast with normal nutritional group, people under malnutrition status tended to have BADL disability (*P* < 0.001), rare or never eat meat (*P* = 0.001), fish (*P* = 0.007), eggs (*P* = 0.015), and vegetables (*P* < 0.001). However, ethnicity, residence, living alone, and history of heart diseases, diabetes, and arthritis were not significantly different across the three groups.

**TABLE 1:** Characteristics of participants.

Characteristics	Total population (*n* = 1,632)	Nutritional status			*P*-value
		Normal (*n* = 1,060)	Mild malnutrition (*n* = 317)	Moderate-to-severe malnutrition (*n* = 255)	
Age (years, mean ± SD)^a^	85.57 ± 12.35	81.58 ± 11.88	90.96 ± 10.35	95.40 ± 7.67	<0.001
Height (cm, mean ± SD)^a^	153.65 ± 11.04	155.18 ± 11.21	151.05 ± 10.34	150.56 ± 9.88	<0.001
Weight (kg, mean ± SD)^a^	51.20 ± 12.58	55.45 ± 11.90	45.50 ± 9.87	40.63 ± 8.76	<0.001
BMI (kg/m^2^, mean ± SD)^a^	21.53 ± 4.03	22.91 ± 3.36	19.87 ± 3.50	17.85 ± 3.03	<0.001
Serum albumin (g/L, mean ± SD)^a^	41.96 ± 4.40	44.13 ± 2.95	39.71 ± 2.82	35.71 ± 3.63	<0.001
Sex^c^, n (%)					<0.001
Male	741 (45.4)	553 (52.2)	111 (35.0)	77 (30.2)	
Female	891 (54.6)	507 (47.8)	206 (65.0)	178 (69.8)	
Ethnicity^c^, n (%)					0.488
Han	1,406 (86.2)	906 (85.5)	275 (86.8)	225 (88.2)	
Other	226 (13.8)	154 (14.5)	42 (13.2)	30 (11.8)	
Residence^c^, n (%)					0.228
Urban	1,582 (96.9)	1,032 (97.4)	307 (96.8)	243 (95.3)	
Rural	50 (3.1)	28 (2.6)	10 (3.2)	12 (4.7)	
Marital status^c^, n (%)					<0.001
Married	648 (39.7)	524 (49.4)	91 (28.7)	33 (12.9)	
Other	984 (60.3)	536 (50.6)	226 (71.3)	222 (87.1)	
Education^c^, n (%)					<0.001
No schooling	1,043 (63.9)	589 (55.6)	239 (75.4)	215 (84.3)	
Some schooling	589 (36.1)	471 (44.4)	78 (24.6)	40 (15.7)	
Living alone c, n (%)					0.289
Yes	345 (21.1)	212 (20.0)	72 (22.7)	61 (23.9)	
No	1,287 (78.9)	848 (80.0)	245 (77.3)	194 (76.1)	
Smoking^c^, n (%)					0.008
Yes	285 (17.5)	207 (19.5)	47 (14.8)	31 (12.2)	
No	1,347 (82.5)	853 (80.5)	270 (85.2)	224 (87.8)	
Drinking^c^, n (%)					0.004
Yes	291 (17.8)	212 (20.0)	49 (15.5)	30 (11.8)	
No	1,341 (82.2)	848 (80.0)	268 (84.5)	225 (88.2)	
Regular exercising^c^, n (%)					0.001
Yes	255 (15.6)	185 (17.5)	49 (15.5)	21 (8.2)	
No	1,377 (84.4)	875 (82.5)	268 (84.5)	234 (91.8)	
Heart disease^c^, n (%)					0.116
Yes	118 (7.2)	87 (8.2)	17 (5.4)	14 (5.5)	
No	1,514 (92.8)	973 (91.8)	300 (94.6)	241 (94.5)	
Hypertension^c^, n (%)					<0.001
Yes	1,006 (61.6)	699 (65.9)	171 (53.9)	136 (53.3)	
No	626 (38.4)	361 (34.1)	146 (46.1)	119 (46.7)	
Diabetes^c^, n (%)					0.177
Yes	31 (1.9)	25 (2.4)	3 (0.9)	3 (1.2)	
No	1,601 (98.1)	1,035 (97.6)	314 (99.1)	252 (98.8)	
Arthritis c, n (%)					0.938
Yes	136 (8.3)	87 (8.2)	28 (8.8)	21 (8.2)	
No	1,496 (91.7)	973 (91.8)	289 (91.2)	234 (91.8)	
BADL disability					<0.001
Yes	265 (16.2)	87 (8.2)	77 (24.3)	101 (39.6)	
No	1,367 (83.8)	973 (91.8)	240 (75.7)	154 (60.4)	
Meat intake					0.001
Almost every day	1,455 (89.2)	961 (90.7)	278 (87.7)	216 (84.7)	
Occasionally	96 (5.9)	61 (5.8)	21 (6.6)	14 (5.5)	
Rarely or never	81 (4.9)	38 (3.5)	18 (5.7)	25 (9.8)	
Fish intake					0.007
Almost every day	1,265 (77.5)	830 (78.3)	243 (76.7)	192 (75.3)	
Occasionally	211 (12.9)	140 (13.2)	47 (14.8)	24 (9.4)	
Rarely or never	156 (9.6)	90 (8.5)	27 (8.5)	39 (15.3)	
Eggs intake					0.015
Almost every day	1,410 (86.4)	936 (88.3)	268 (84.5)	206 (80.8)	
Occasionally	103 (6.3)	58 (5.5)	25 (7.9)	20 (7.8)	
Rarely or never	119 (7.3)	66 (6.2)	24 (7.6)	29 (11.4)	
Vegetable intake					<0.001
Almost every day	1,416 (86.8)	947 (89.3)	268 (84.5)	201 (78.8)	
Occasionally	165 (10.1)	90 (8.5)	38 (12.0)	37 (14.5)	
Rarely or never	51 (3.1)	23 (2.2)	11 (3.5)	17 (6.7)	
MMSE score in 2011–2012 wave^b^, median (P_25_, P_75_)	28.0 (21.0, 29.0)	28.0 (25.0, 30.0)	25.0 (15.0, 29.0)	20.0 (11.0, 28.0)	<0.001
MMSE score in 2014 wave^b^, median (P_25_, P_75_)	27.0 (21.0, 29.0)	28.0 (24.0, 30.0)	23.0 (12.0, 27.0)	20.0 (6.0, 26.0)	<0.001
MMSE score in 2017–2018 wave^b^, median (P_25_, P_75_)	28.0 (23.0, 29.0)	28.0 (25.0, 29.0)	24.0 (15.0, 28.0)	24.0 (18.5, 28.5)	<0.001

*BMI, body mass index; BADL, basic activities of daily living; MMSE, Mini-Mental State Examination.*

*^a^These variables were analyzed using one-way ANOVA.*

*^b^These variables were analyzed using the Kruskal–Wallis non-parametric test.*

*^c^These variables were analyzed using the χ^2^ test.*

### Association Between Malnutrition and Cognitive Function

Table 2 shows the association between malnutrition and cognitive function in the total population. Whether adjusting for covariates or not, people in mild and moderate-to-severe malnutrition groups have a poorer cognitive function (adjusted *P* = 0.007 and *P* < 0.001, respectively). Compared with the normal nutrition group, those with mild and moderate-to-severe malnutrition have a lower MMSE score of 0.95 and 1.39 points (adjusted β = –0.95, 95% *CI* = –1.60 to –0.25; adjusted β = –1.39, 95% *CI* = –2.21 to –0.57, respectively). Moreover, the MMSE score has shown a significant linear downward trend with increasing malnutrition degrees (adjusted *P* for trend < 0.001).

**TABLE 2:** The association of malnutrition with cognitive function.

Nutrition status	Crude			Adjusted*		
	β	*P*	95% CI	β	*P*	95% CI
Normal (*n* = 1,060)	Ref	Ref	Ref	Ref	Ref	Ref
Mild (*n* = 317)	–4.84	<0.001	–5.57 to –4.10	–0.95	0.007	–1.60 to –0.25
Moderate–to–severe (*n* = 255)	–7.23	<0.001	–8.10 to –6.36	–1.39	<0.001	–2.21 to –0.57
*P* for trend	–3.88	<0.001	–4.28 to –3.48	–0.74	<0.001	–1.13 to –0.35

*In the adjusted model, age, sex, ethnicity, residence, marital status, education, living alone, regular exercising, smoking status, drinking status, heart disease history, diabetes history, hypertension history, arthritis history, BADL disability, and food intake frequency of vegetables, meat, eggs, and fish were adjusted.

Table 3 shows the relationship between malnutrition and cognitive function stratified by sex. In males, after adjusting for covariates, a significant association was not found in both mild and moderate-to-severe malnutrition groups (adjusted *P* = 0.196 and *P* = 0.514, respectively). While for female subjects, there was a negative association of mild and moderate-to-severe malnutrition with cognitive function regardless of adjusting covariates or not (adjusted *P* = 0.024, β = –1.12, and 95% *CI* = –2.08 to –0.15; and *P* = 0.001, β = –1.94, and 95% *CI* = –3.09 to –0.79, respectively). A significant trend of a cognitive decline with increasing malnutrition degrees was observed in females (adjusted *P* for trend < 0.001) but not in males (adjusted *P* for trend = 0.289).

**TABLE 3:** The association of malnutrition with cognitive function stratified by sex.

Subgroups	Crude			Adjusted*		
	β	*P*	95% CI	β	*P*	95% CI
**Males (*n* = 741)**						
Normal	Ref	Ref	Ref	Ref	Ref	Ref
Mild	–3.23	<0.001	–4.19 to –2.26	–0.61	0.196	–1.53 to 0.31
Moderate-to-severe	–4.30	<0.001	–5.51 to –3.11	–0.39	0.514	–1.55 to 0.78
*P* for trend	–2.42	<0.001	–2.96 to –1.89	–0.29	0.289	–0.83 to 0.25
**Females (*n* = 891)**						
Normal	Ref	Ref	Ref	Ref	Ref	Ref
Mild	–4.83	<0.001	–5.88 to –3.78	–1.12	0.024	–2.08 to –0.15
Moderate-to-severe	–7.70	<0.001	–8.90 to –6.50	–1.94	0.001	–3.09 to –0.79
*P* for trend	–4.03	<0.001	–4.60 to –3.46	–0.99	<0.001	–1.55 to –0.44

*In the adjusted model, age, ethnicity, residence, marital status, education, living alone, regular exercising, smoking status, drinking status, heart disease history, diabetes history, hypertension history, arthritis history, BADL disability, and food intake frequency of vegetables, meat, eggs, and fish were adjusted.

The association between malnutrition and cognitive function stratified by age is presented in Table 4. After adjusting for covariates, there was no association of mild and moderate-to-severe malnutrition with cognitive function among the elderly aged 60–79 years (adjusted *P* = 0.057 and *P* = 0.821, respectively) and 80–89 years (adjusted *P* = 0.695 and *P* = 0.490, respectively). While among the elderly above 90 years old, a significant association was observed between mild and moderate-to-severe malnutrition and cognitive function (adjusted *P* = 0.003, β = –2.04, and 95% *CI* = –3.37 to –0.71; and *P* < 0.001, β = –2.54, and 95% *CI* = –3.93 to -1.15, respectively). Moreover, a linear trend in the association of malnutrition risk with cognitive performance was also found in individuals aged 90 years and above (adjusted *P* for trend < 0.001).

**TABLE 4:** The association of malnutrition with cognitive function stratified by age.

Subgroups	Crude			Adjusted*		
	β	*P*	95% CI	β	*P*	95% CI
**60–79 years old (*n* = 515)**						
Normal	Ref	Ref	Ref	Ref	Ref	Ref
Mild	–1.01	0.012	–1.79 to –0.22	–0.69	0.057	–1.40 to 0.02
Moderate-to-severe	–0.32	0.722	–2.09 to 1.45	–0.18	0.821	–1.77 to 1.40
*P* for trend	–0.63	0.037	–1.23 to 0.04	–0.51	0.093	–1.11 to 0.09
**80-89 years old (*n* = 420)**						
Normal	Ref	Ref	Ref	Ref	Ref	Ref
Mild	–0.75	0.176	–1.84 to 0.34	–0.21	0.695	–1.29 to 0.86
Moderate-to-severe	–1.02	0.162	–2.46 to 0.41	–0.51	0.490	–1.95 to 0.93
*P* for trend	–0.58	0.077	–1.21 to 0.06	–0.26	0.428	–0.91 to 0.39
**≥90 years old (*n* = 697)**						
Normal	Ref	Ref	Ref	Ref	Ref	Ref
Mild	–3.30	<0.001	–4.68 to –1.91	–2.04	0.003	–3.37 to –0.71
Moderate-to-severe	–4.39	<0.001	–5.84 to -2.95	–2.54	<0.001	–3.93 to –1.15
*P* for trend	–2.30	<0.001	–3.01 to –1.58	–1.33	<0.001	–2.03 to –0.63

*In the adjusted model, sex, ethnicity, residence, marital status, education, living alone, regular exercising, smoking status, drinking status, heart disease history, diabetes history, hypertension history, arthritis history, BADL disability, and food intake frequency of vegetables, meat, eggs, and fish were adjusted.

After adjusting for covariates, a significant association and trend between malnutrition and cognitive impairment were found for the illiterate Chinese elderly (adjusted *P* = 0.004 and 0.007 for mild and moderate-to-severe malnutrition, respectively; adjusted *P* for trend = 0.002), while the association disappeared among the elderly who ever attended school (Table 5).

**TABLE 5:** The association of malnutrition with cognitive function stratified by education level.

Subgroups	Crude			Adjusted*		
	β	*P*	95% CI	β	*P*	95% CI
**No schooling (*n* = 1,043)**						
Normal	Ref	Ref	Ref	Ref	Ref	Ref
Mild	-4.87	<0.001	–5.85 to –3.88	–1.35	0.004	–2.26 to –0.43
Moderate-to-severe	–6.47	<0.001	–7.58 to –5.36	–1.45	0.007	–2.50 to –0.40
*P* for trend	–3.52	<0.001	–4.04 to –2.99	–0.82	0.002	–1.32 to –0.31
**Schooling (*n* = 589)**						
Normal	Ref	Ref	Ref	Ref	Ref	Ref
Mild	–1.56	<0.001	–2.45 to –0.67	0.20	0.663	–0.69 to 1.08
Moderate-to-severe	–4.56	<0.001	–5.84 to –3.29	–1.16	0.080	–2.46 to 0.14
*P* for trend	–2.05	<0.001	–2.59 to –1.51	–0.34	0.250	–0.91 to 0.24

*In the adjusted model, age, sex, ethnicity, residence, marital status, living alone, regular exercising, smoking status, drinking status, heart disease history, diabetes history, hypertension history, arthritis history, BADL disability, and food intake frequency of vegetables, meat, eggs, and fish were adjusted.

### Sensitivity Analysis

As shown in Supplementary Table 2, there is a significant positive relationship of the continuous GNRI and serum albumin with cognitive function, regardless of adjusting or not (crude and adjusted *P* < 0.001). Namely, subjects with a higher GNRI or serum albumin have a better cognitive function, while BMI was not associated with cognitive decline (adjusted *P* = 0.499). In the mediation analysis, as shown in Supplementary Table 3, hypertension, rather than diabetes, had a significant partially mediating effect on the relationship between malnutrition and cognitive impairment. The mediating effect was –0.095 (–0.164 to –0.025), and the proportion mediated of hypertension was 9.65%. Moreover, as shown in Supplementary Table 4, a significant association remained between mild (adjusted *P* = 0.024) and moderate-to-severe (adjusted *P* = 0.004) malnutrition and cognitive function in the complete dataset.

## Discussion

A longitudinal study of 1,632 Chinese elderly was used to explore the relationship between malnutrition and cognitive function. We found that malnutrition was associated with cognitive function. Furthermore, subjects with more serious malnutrition have a poorer cognitive performance, especially in illiterate females and the oldest elderly. With increasing degrees of malnutrition, the cognitive function has shown a worsening trend.

Participants were classified into different nutrition status groups according to the GNRI. Notably, 19.4% of participants were under mild malnutrition risk, and 15.6% were moderate-to-severe malnourished. People with malnutrition status were older than those with well-nourished status. This result is in line with studies using alternative methods, such as MNA or MNA-SF, to assess the elderly malnutrition status (Mantzorou et al., 2020; Yu et al., 2021). With the increasing degrees of malnutrition, the MMSE score decreased with a linear trend, which indicated that the cognitive function has become worsening. This is consistent with the finding of a recent study, which revealed that severe nutritional status was related to a faster cognitive decline (Sanders et al., 2016).

Serum albumin, as an indicator to assess nutritional status and inflammation level, has been used to predict adverse clinical outcomes (Don and Kaysen, 2004; Abubakar et al., 2013). Recent studies have shown that a higher albumin level was associated with a lower risk of cognitive impairment (Llewellyn et al., 2010; Yin et al., 2016; Wang et al., 2018). Our results were comparable. Evidence suggested that serum albumin can combine with amyloid-β and can reduce its neurotoxicity by inhibiting its aggregation and fibrosis, thus preventing further progression of cognitive decline (Stanyon and Viles, 2012). Meanwhile, it is reported that BMI has an effect on energy metabolism through regulating body cell mass, which is one of the key factors to reflect nutritional status (Oliveira et al., 2020). According to previous studies, overweight old people were found to have a lower risk of cognitive impairment or dementia compared with under or normal weight (Hou et al., 2019; Li et al., 2020). However, in our study, we found that BMI was not significantly associated with cognitive function. In fact, BMI may not be a useful tool to assess malnutrition among elders. It did not take into account the distribution of body fat and could not classify lean body mass and fat mass in the elderly (Goyal et al., 2014). A higher risk of cognitive decline in elders with a low BMI may be due to obesity with low muscle mass and increased fat (Riobó Serván et al., 2015). While the GNRI is based on serum albumin and weight loss, which was not selfsame with BMI. Furthermore, a higher weight was given for albumin than for weight according to the formula of the GNRI. Therefore, a significant association of malnutrition with cognitive function was reasonable.

In this study, a strong association between malnutrition and cognitive function was found among individuals aged 90 years and above. We speculated that there was a similar increasing trend of malnutrition risk and cognitive decline with aging, which caused this association. On the one hand, older age has been identified as a risk factor of malnutrition (Guyonnet and Rolland, 2015). Also, previous studies showed that with every 1-year increase in age, the risk of being under malnourished would increase by 8.5% (Wei et al., 2018). On the other hand, it was documented that more than half of the individuals above 60 years old would have a deteriorating cognitive function with aging. Also, the magnitude of the decline in cognitive function was greater in the older elderly (Davis et al., 2018; Zhang et al., 2019).

In addition, a significant association also appeared in female individuals and participants without attending school. A possible explanation might be due to socioeconomic disadvantages, lacking education, and worse financial situation. As reported earlier, females were more likely to have a higher prevalence of malnutrition and cognitive impairment (Au et al., 2017; Ning et al., 2021). Meanwhile, education attainment, as a proxy for cognitive reserve (Staekenborg et al., 2020), was positively associated with the level of the executive function and episodic memory in later life (Liu and Lachman, 2020). Also, participants with a higher educational level would have better nutritional intakes to reduce malnutritional risk (Rippin et al., 2020).

Therefore, although the underlying mechanism of the relationship between malnutrition and cognitive function is not clear until present, the GNRI defined that using serum albumin and weight loss has been validated in exploring the association of malnutrition with cognition function in this study. Researchers should make full use of the GNRI, rather than a single biomarker, in further malnutrition-related studies.

### Strengths and Limitations

Our study has some strengths. First, because the CLHLS is a representative study in the Chinese elderly, the conclusion of our current study was more credible and reproducible. Second, compared with the previous study, we used the CLHLS, a longitudinal cohort study, to examine the association between malnutrition and cognitive function, which makes it easier to interpret the causal link between malnutrition and cognitive function. Finally, to our knowledge, the GNRI was rarely used to evaluate the relationship between malnutrition and cognitive function, particularly in the Chinese elderly. Compared to another single indictor, the GNRI has been considered as a reliable comprehensive tool for assessing malnutrition in the elderly. Thus, our study could provide solid evidence on the association of the GNRI with cognitive impairment, which is beneficial for delaying or preventing the occurrence and progression of cognitive impairment. However, this study had some limitations. First, since the CLHLS was designed for the oldest-old Chinese, generalizing the results to other populations should be cautioned. Second, although we collected confounding factors such as smoking, drinking, food intake frequency, hypertension, and history of diabetes, it may lead to a potential recall bias as some of them were self-reported. Third, since biomarkers were assayed for once in the random sample, the changes over time of biomarkers failed to be considered in this study. Finally, this study was enriched for octogenarians and nonagenarians, who might be healthier and less likely to suffer from malnutrition and cognitive impairment. Therefore, this study might underestimate the association of malnutrition with cognitive function.

## Conclusion

A significant association of malnutrition measured by the GNRI with cognitive performance was observed among the Chinese elderly. Subjects with malnutrition have a poorer cognitive function, especially in illiterate females and the oldest elderly. Furthermore, the cognitive function was worse with malnutrition aggravating. Accordingly, the GNRI should be used broadly to evaluate and improve the nutritional status of the targeted elderly population. Moreover, improving early detection of malnutrition and managing routine nutritional surveillance are crucial for the elderly to prevent cognitive impairment.

## Data Availability Statement

The datasets presented in this study can be found in online repositories: The CLHLS questionnaires are available at https://sites.duke.edu/centerforaging/programs/chinese-longitudinal-healthy-longevity-survey-clhls/surveydocumentation/questionnaires/ and the full datasets used in this analysis are available at https://opendata.pku.edu.cn/dataverse/CHADS.

## Ethics Statement

This study was approved by the Institutional Review Board, Duke University (Pro00062871), and the Biomedical Ethics Committee, Peking University (IRB00001052–13074). The patients/participants provided their written informed consent to participate in this study.

## Author Contributions

YC: conceptualization, formal analysis, and supervision. WL and YC: methodology, review, and editing. BS and YZ: writing–original draft preparation. All authors read and approved the final version of the manuscript.

## Conflict of Interest

The authors declare that the research was conducted in the absence of any commercial or financial relationships that could be construed as a potential conflict of interest.

## Publisher’s Note

All claims expressed in this article are solely those of the authors and do not necessarily represent those of their affiliated organizations, or those of the publisher, the editors and the reviewers. Any product that may be evaluated in this article, or claim that may be made by its manufacturer, is not guaranteed or endorsed by the publisher.
