# Supplementary material for: The Relationship of Malnutrition With Cognitive Function in the Older Chinese Population: Evidence From the Chinese Longitudinal Healthy Longevity Survey Study
Source: Front Aging Neurosci. 2021 Nov 22;13:766159. doi: 10.3389/fnagi.2021.766159 (PMC8645828; doi:10.3389/fnagi.2021.766159)
Supplement: Supplementary file 1 [file Table_1.docx]

**Supplementary Table 1 The Chinese version of the mini-mental state examination in the CLHLS**

| Domains | Questions | Scores |
| --- | --- | --- |
| Orientation | What time of day is it right now (morning, afternoon, evening)? | 1 |
|  | What is the month (Western or Chinese calendar) right now? | 1 |
|  | What is the date (Chinese calendar day and month) of the mid-autumn festival? | 1 |
|  | What is the season right now, spring, summer, fall, winter? | 1 |
|  | What is the name of this district or town? | 1 |
|  | Please name as many kinds of food as possible in 1 minute. | 7 |
| Registration | repeat table, apple and clothes | 3 |
| Attention and calculation | I will ask you to spend 3 dollars from 20 dollars, then you must spend 3 dollars from the number you arrived at and continue to  spend 3 dollars until you are asked to stop. | 5 |
|  | Asking the interviewee to copy a figure, in which all the sides and angles are correct | 1 |
| Recall | repeat the three words (in any order) that you heard a little while ago | 3 |
| Language | Naming pen and watch. | 2 |
|  | Repeating the following sentence: “What you plant, what you will get.” | 1 |
|  | The individual is asked to take a paper using right hand, fold it in the middle using both hands, and place the paper on the floor. | 3 |

**Supplementary Table 2 Sensitivity analysis of the associations of the GNRI as a continuous variable, serum albumin, and BMI with cognitive function**

| **Models** | Crude | | |  | Adjusted^*^ | | |
| --- | --- | --- | --- | --- | --- | --- | --- |
|  | *β* | *P* | *95%CI* |  | *β* | *P* | *95%CI* |
| **Model 1** |  |  |  |  |  |  |  |
| GNRI | 0.34 | <0.001 | 0.31~ 0.38 |  | 0.06 | 0.001 | 0.02~ 0.09 |
| **Model 2** |  |  |  |  |  |  |  |
| Serum albumin | 0.59 | <0.001 | 0.53~ 0.66 |  | 0.11 | 0.001 | 0.04~ 0.17 |
| **Model 3** |  |  |  |  |  |  |  |
| BMI | 0.37 | <0.001 | 0.30~ 0.44 |  | 0.02 | 0.499 | -0.04~ 0.08 |

^*^In the adjusted model, age, sex, ethnicity, residence, marital status, education, living alone, regular exercising, smoking status, drinking status, heart disease history, diabetes history, hypertension history, [arthritis](javascript:;) history, basic activities of daily living (BADL) disability, and food frequency intake of vegetable, meat, eggs, and fish were adjusted.

**Supplementary Table 3. Mediating effect of hypertension and diabetes on the associations of malnutrition with cognitive function**

| Paths | *β* | *P* | Bootstrap 95% *CI* | Proportions mediated  (%) ^†^ |
| --- | --- | --- | --- | --- |
| **Hypertension as a mediator^※^** |  |  |  |  |
| Total effect (path c) | -0.981 | < 0.001 | -1.460~ -0.501 |  |
| Direct effect (path c’) | -0.886 | < 0.001 | -1.371~-0.401 |  |
| Indirect effect | -0.095 | 0.008 | -0.164~-0.025 | 9.65 |
| **Diabetes as a mediator^＃^** |  |  |  |  |
| Total effect (path c) | -0.887 | 0.025 | -1.661~ -0.113 |  |
| Direct effect (path c’) | -0.886 | < 0.001 | -1.371~ -0.401 |  |
| Indirect effect | -0.001 | 0.833 | -0.008~ 0.006 | 0.08 |

^※^ adjusted for age, sex, ethnicity, residence, marital status, education, living alone, regular exercising, smoking status, drinking status, heart disease history, diabetes history, [arthritis](javascript:;) history, basic activities of daily living (BADL) disability, and food frequency intake of vegetable, meat, eggs, and fish

^＃^ adjusted for age, sex, ethnicity, residence, marital status, education, living alone, regular exercising, smoking status, drinking status, heart disease history, hypertension history, [arthritis](javascript:;) history, basic activities of daily living (BADL) disability, and food frequency intake of vegetable, meat, eggs, and fish.

^†^The mediated proportion was calculated as indirect effect divided by total effect.

**Table 4. The association of malnutrition with cognitive function using multiple imputation dataset (n=2064)**

| Nutrition status | Crude | | |  | Adjusted^*^ | | |
| --- | --- | --- | --- | --- | --- | --- | --- |
|  | *β* | *P* | *95%CI* |  | *β* | *P* | *95%CI* |
| Normal (n=1363) | *Ref* | *Ref* | *Ref* |  | *Ref* | *Ref* | *Ref* |
| Mild (n=389) | -4.63 | <0.001 | -5.47~ -3.80 |  | -0.99 | 0.024 | -1.81~ -0.17 |
| Moderate-to-severe (n=312) | -7.72 | <0.001 | -8.66~ -6.77 |  | -1.83 | 0.004 | -2.91~ -0.75 |
| *P* for trend | -3.99 | <0.001 | -4.44~ -3.54 |  | -0.93 | 0.004 | -1.46~ -0.39 |

^*^In the adjusted model, age, sex, ethnicity, residence, marital status, education, regular exercising, smoking status, drinking status, diabetes history, hypertension history, basic activities of daily living (BADL) disability, and food frequency intake of vegetable, meat, eggs, and fish were adjusted.
